# Exploring the significance of PAK1 through chromosome conformation signatures in ibrutinib‐resistant chronic lymphocytic leukaemia

**DOI:** 10.1002/1878-0261.13281

**Published:** 2022-07-22

**Authors:** Zijuan Wu, Luqiao Wang, Lei Fan, Hanning Tang, Xiaoling Zuo, Danling Gu, Xueying Lu, Yue Li, Jiazhu Wu, Shuchao Qin, Yi Xia, Huayuan Zhu, Li Wang, Wei Xu, Jianyong Li, Hui Jin

**Affiliations:** ^1^ Department of Hematology, Pukou CLL Center, Jiangsu Province Hospital The First Affiliated Hospital of Nanjing Medical University China; ^2^ Key Laboratory of Hematology of Nanjing Medical University China; ^3^ Jiangsu Key Lab of Cancer Biomarkers, Prevention and Treatment, Collaborative Innovation Center for Personalized Cancer Medicine Nanjing Medical University China; ^4^ National Clinical Research Center for Hematologic Diseases The First Affiliated Hospital of Soochow University Suzhou China

**Keywords:** chronic lymphocytic leukaemia, ibrutinib resistance, metabolism, PAK1, three‐dimensional genome

## Abstract

Ibrutinib exerts promising anticancer effects in chronic lymphocytic leukaemia (CLL). However, acquired resistance occurs during treatment, necessitating the exploration of underlying mechanisms. Although three‐dimensional genome organization has been identified as a major player in the development and progression of cancer, including drug resistance, little is known regarding its role in CLL. Therefore, we investigated the molecular mechanisms underlying ibrutinib resistance through multi‐omics analysis, including high‐throughput chromosome conformation capture (Hi‐C) technology. We demonstrated that the therapeutic response to ibrutinib is associated with the expression of p21‐activated kinase 1 (PAK1). PAK1, which was up‐regulated in CLL and associated with patients' survival, was involved in cell proliferation, glycolysis and oxidative phosphorylation. Furthermore, the PAK1 inhibitor IPA‐3 exerted an anti‐tumour effect and its combination with ibrutinib exhibited a synergistic effect in ibrutinib‐sensitive and ‐resistant cells. These findings suggest the oncogenic role of PAK1 in CLL progression and drug resistance, highlighting PAK1 as a potential diagnostic marker and therapeutic target in CLL including ibrutinib‐resistant CLL.

## Graphical abstract

The oncogene PAK1 was found located in a region where B‐to‐A compartment switching occurred in established ibrutinib‐resistant CLL cells. PAK1 regulated the oxidative phosphorylation and glycolysis of CLL cells, promoted cell proliferation and affected the sensitivity to ibrutinib.

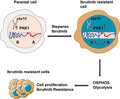

**Abbreviations**
3D: three‐dimensional
ATAC: assay for transposase‐accessible chromatin
BTK: Bruton's tyrosine kinase
CI: combination index
CLL: chronic lymphocytic leukaemia
ECAR: extracellular acidification rate
GSEA: gene set enrichment analysis
Hi‐C: high‐throughput chromosome conformation capture
MEC‐1R: ibrutinib‐resistant MEC‐1 cells
OCR: oxygen consumption rate
OS: overall survival
PAK1: p21‐activated kinase 1
PBMC: peripheral blood mononuclear cell
RT‐qPCR: real‐time quantitative PCR
STR: short tandem repeat
TAD: topologically associating domains
TMT: tandem mass tag

## 1 Introduction

Chronic lymphocytic leukaemia (CLL) is a lymphoproliferative disorder that mainly affects the elderly, characterized by the expansion of small mature B‐cells [1]. New targeted drugs have greatly improved CLL patients' survival. Ibrutinib, as the first FDA‐approved Bruton's tyrosine kinase (BTK) inhibitor, is widely applied to first/second‐line treatment in patients with CLL. However, drug resistance happens during the treatment process which has not been well understood [2, 3]. Bruton's tyrosine kinase and PLCγ2 mutations are most frequently described mutations that confer resistance to ibrutinib. And they were detectable in approximately 85% of patients with CLL who relapsed after treatment with ibrutinib [4]. Besides primary resistance, about 8–13% of acquired resistance happened in CLL patients [5]. However, the underlying molecular mechanisms are yet not well‐known especially for those patients without mutations. Therefore, it is important to explore the molecular mechanisms contributing to ibrutinib resistance in CLL.

The three‐dimensional (3D) spatial structure of chromatin is highly dynamic and varies greatly between cell types and developmental stages. Chromatin homeostasis plays a major role in disease prevention [6], as changes in the 3D genomic structure can impact stem cell development and differentiation, disease progression and drug resistance [7, 8]. Accordingly, alterations in the genomic spatial structure and chromatin interactions have recently emerged as new therapeutic targets [9, 10]. Mas et al. [11] reported the key roles of the chromatin architecture in the fate of stem cells. Furthermore, Barutcu et al. [12] confirmed that switching chromatin compartments could contribute to gene dysregulation and thus promote disease progression, using high‐throughput chromosome conformation capture (Hi‐C) approach. Wu et al. [13] reported alterations at multiple levels, including spatial genome reorganization, in multiple myeloma, and the significance of genomic stability maintenance was also revealed in CLL [14]. Therefore, we hypothesized that the alteration of chromatin conformation is involved in the resistance of CLL to ibrutinib. However, the underlying mechanism remains unclear.

Given that genome architecture is pivotal in regulating biological processes, we sought to explore alterations in the 3D chromatin organization between ibrutinib‐sensitive and ‐resistant CLL cells. Particularly, MEC‐1 cells are Epstein–Barr virus‐positive CLL cells with a TP53 mutation, complex karyotype and overexpression of Bcl‐2, and are thus able to mimic the characteristics of CLL patients who are at high risk of developing resistance to ibrutinib [15]. We used an integrated multi‐omics approach with ibrutinib‐resistant cells established from MEC‐1 cells to clarify the changes of chromatin spatial organization and identify the oncogenic factors contributing to ibrutinib resistance. This study can therefore offer valuable guidance towards identifying potential therapeutic targets for CLL.

## 2 Materials and methods

### 2.1 Human samples and cell lines

Anticoagulated (with ethylenediaminetetraacetic acid) peripheral blood samples were obtained from CLL patients and healthy volunteers at the First Affiliated Hospital of Nanjing Medical University, Jiangsu Province Hospital (Nanjing, China). And the experiments were undertaken with the understanding and written consent of each subject. The use of human samples was approved by the institutional ethics committee (Approval Number 2021‐SRFA‐208). Peripheral blood mononuclear cells (PBMCs) were isolated via density gradient centrifugation with Lymphoprep™ (Stemcell Technologies, Vancouver, Canada) to generate single‐cell suspensions. The study methodologies conformed to the standards set by the Declaration of Helsinki.

The CLL cell lines MEC‐1 and JVM‐3 were purchased from Cobioer (Nanjing, China) and cultured in RPMI‐1640 medium (Gibco, Grand Island, NY, USA) supplemented with 10% foetal bovine serum (Gibco) and 100 μg·mL^−1^ penicillin/streptomycin (Gibco) at 37 °C in a humidified atmosphere (5% CO_2_). The cell lines used in this study were authenticated via short tandem repeat (STR) profiling. Ibrutinib‐resistant MEC‐1 cells (MEC‐1R) were established by exposure to 1 μm ibrutinib. Following recovery to a normal growth rate, the cells were continuously treated with increasing doses of ibrutinib (3, 5, 7, 10, 15 and 20 μm) over the course of approximately 6 months. Resistance was confirmed through cell proliferation and cell apoptosis assay.

### 2.2 Lentiviral transduction

Lentiviral constructs for stable *PAK1* overexpression (#LV‐68071–1) and RNA interference (RNAi) targeting *PAK1* (#100389‐1, #100390‐1, #100391‐1) were purchased from GENECHEM (Shanghai, China) as viral particles. Virus infection of various cell lines was performed according to the manufacturer's instructions. The concentrated lentivirus stock and HiTransB‐2 were added to 200 000 cells and spun at 200 ***g*** for 60 min. After 48 h, the transduced cells were selected with 5 μg·mL^−1^ puromycin and the transfection efficiency was determined via real‐time quantitative PCR (RT‐qPCR) and western blot.

### 2.3 RNA isolation and RT‐qPCR

Total RNA was isolated using TRIzol reagent (Life Technologies, Carlsbad, CA, USA) according to the manufacturer's instructions and reverse‐transcribed using HiScript reverse transcriptase (Vazyme, Nanjing, China). qPCR was then performed using Hieff qPCR SYBR Green master mix (Yeasen, Shanghai, China), with *GAPDH* as a housekeeping gene. Primer sequences were as follows: *PAK1*, GTGAAGGCTGTGTCTGAGACTC (forward primer) and GGAAGTGGTTCAATCACAGACCG (reverse primer); *BCAT1*, GCTCTGGTACAGCCTGTGTTGT (forward primer) and TGCCAGCTTAGGACCATTCTCC (reverse primer); *GAPDH*, CTGGGCTACACTGAGCACC (forward primer) and AAGTGGTCGTTGAGGGCAATG (reverse primer).

### 2.4 Western blotting

Preparation of whole‐cell protein lysates and western blot analysis were performed as described previously [16]. Primary antibodies against PAK1 (2602), p‐PAK1 (2601), p70 S6 kinase (2708), p‐P70S6K (Thr421/Ser424; 9240), 4E‐BP1 (9452) and p‐4E‐BP1 (Thr37/46; 2855) were obtained from Cell Signaling Technology (Danvers, MA, USA). The antibody against BCAT1 (ab232700) was purchased from Abcam (Cambridge, UK) and the β‐actin (AF0003) antibody (as a control) was obtained from Beyotime (Shanghai, China).

### 2.5 Cell proliferation and growth inhibition assays

Cell proliferation was assessed using Cell Counting Kit‐8 (CCK8; APExBIO, Houston, TX, USA). Briefly, the cells were seeded in 96‐well plates and incubated with CCK8 solution (10 μL per well) for 2 h; the absorbance was then measured at 450 nm. For growth inhibition analysis, cells were simultaneously incubated with ibrutinib (Selleck Chemicals, S2680, Shanghai, China), IPA‐3 (Selleck Chemicals, S7093), gabapentin (Selleck Chemicals, S2133), BCATc inhibitor 2 (APExBIO, C3463) or a combination of these agents. The synergistic effect of the drugs was determined using the combination index (CI) parameter, wherein a CI value of < 1 indicates synergism, CI = 1 indicates additivity and CI > 1 indicates antagonism [17]. Quantitative analysis of data was conducted using calcusyn version 2.0 software (Biosoft, Cambridge, UK).

### 2.6 Apoptosis assay

Cells were collected and stained using Annexin V‐FITC/propidium iodide (PI; FcMACS, Nanjing, China) as described previously [18]. After incubation in the dark for 15 min, apoptosis was detected via flow cytometry (Cytoflex, Beckman Coulter, Brea, CA, USA).

### 2.7 Seahorse assay

The oxygen consumption rate (OCR) and extracellular acidification rate (ECAR) were measured using Seahorse XFe96 Analyzer (Seahorse Bioscience, Agilent Technologies, Santa Clara, CA, USA). Briefly, 50 000 cells per well were adhered to XF 96‐well cell culture microplates precoated with Cell‐Tak via centrifugation at 200 × ***g*** for 2 min, followed by incubation at 37 °C in a non‐CO_2_ incubator for 20 min. Oligomycin (1 μm), carbonyl cyanide‐4‐(trifluoromethoxy) phenylhydrazone (2 μm) and rotenone/antimycin A (0.5 μm) were loaded into the drug delivery ports and sequentially injected. After each injection, recordings were obtained at four time points with 35‐min intervals. For the glycolysis stress test, glucose, oligomycin and 2‐deoxyglucose were injected at final concentrations of 10 mm, 1 μm and 50 mm respectively.

### 2.8 High‐throughput chromosome conformation capture (Hi‐C), assay for transposase‐accessible chromatin with high‐throughput sequencing (ATAC‐seq), tandem mass tag (TMT) experiments and RNA sequencing

For Hi‐C experiments, MEC‐1 cells were cross‐linked with 1% formaldehyde for 10 min. A 2.5 m glycine solution was then added to a final concentration of 0.2 m and left to react for 15 min. The Hi‐C experiment was performed following a protocol described previously [19]. To obtain valid and normalized chromatin interactions, we used HiC‐Pro [20] (version 2.11.4) to process Hi‐C clean FASTQ data with default parameters, except for MobI restriction sites. All reads were aligned to the UCSC hg19 human genome reference using bowtie2 (version 2.3.0). Valid interactions were then used to generate genome‐wide interaction maps at 100, 50, 10 and 5 kb for the subsequent analyses. Significant interactions (*P* < 0.05) were identified by fithic [21] using HiC‐Pro raw counts. After calling normalized Hi‐C contacts, the comprehensive HiCExplorer tool [22] was used to analyse A/B compartments and topologically associating domains (TADs) and for data visualization. Other analyses and visualization were mainly carried out with custom R programs.

For ATAC‐seq experiments, 50 000 cells were collected and centrifuged at 500 × ***g*** for 5 min, followed by a wash with cold phosphate‐buffered saline. The cells were then lysed with lysis buffer and the supernatant was discarded after centrifugation. The pellet was immediately resuspended in the transposase reaction mix [25 μL 2× TD buffer, 2.5 μL Transposase (Illumina) and 22.5 μL of nuclease‐free water]. The transposition reaction was carried out for 30 min at 37 °C. Directly following transposition, the sample was purified using a Qiagen MinElute kit (Shanghai, China). Following purification, the library fragments were amplified by polymerase chain reaction (PCR) using 1× NEBnext PCR master mix and 1.25 μm of custom Nextera PCR primers under the following thermal programme: 72 °C for 5 min; 98 °C for 30 s; followed by thermocycling at 98 °C for 10 s, 63 °C for 30 s and 72 °C for 1 min; with a final extension at 72 °C for 5 min. The libraries were purified using a Qiagen PCR cleanup kit, yielding a final library [23]. After obtaining the raw data, high‐quality mapped reads (MPAQ ≥ 30) were used for subsequent data analysis after filtering, decontamination and mapping to the reference genome.

For TMT experiments, SDT buffer (sodium dodecyl sulphate, Tris/HCl and dithiothreitol) was added to cells and the lysate was boiled for 15 min. After centrifugation at 14 000 × ***g*** for 40 min, the protein content of the supernatant was quantified using a bicinchoninic acid protein assay kit (P0012, Beyotime). TMT labelling and subsequent procedures were performed as described previously [23]. Tandem mass spectrometry (MS/MS) raw files were processed using the MASCOT engine (Matrix Science, London, UK; version 2.6), input to Proteome Discoverer 2.2 and searched against the UniProt database (http://www.uniprot.org). The search parameters included trypsin as the enzyme used to generate peptides with a maximum of two missed cleavages permitted. A precursor mass tolerance of 10 ppm and a 0.05 Da tolerance for MS2 fragments were specified. In addition to TMT labels, carbamidomethyl (C) was set as a fixed modification. Variable modifications included oxidation (M) and acetylation (at the protein N‐terminus). A peptide and protein false discovery rate of 1% was enforced using a reverse database search strategy. Proteins with a fold change > 1.2 and *P* < 0.05 in Student's *t* test were considered to be differentially expressed. Other analysis and visualization were performed using custom R scripts.

For RNA sequencing, total RNA was extracted from the cells followed by rRNA depletion. The RNA was reverse transcribed to cDNA and constructed into a strand‐specific library after purification by rRNA depletion. Sequencing was performed on an Illumina Novaseq 6000 platform (Illumina, San Diego, CA, USA).

### 2.9 Statistical analysis

Statistical analyses were carried out using graphpad prism 8 (La Jolla, CA, USA). All experiments were performed with three biological replicates and data are expressed as the mean ± SD. Comparisons between two groups were analysed via the two‐tailed Student's *t* test. Differences among multiple groups were analysed via one‐way analysis of variance with *post hoc* comparison using the Tukey's test. The optimal cutoff value of the expression of PAK1 and BCAT1 was determined by ‘surv_cutpoint’ in the r package ‘survminer’. Kaplan–Meier survival curves were analysed by the log rank test. The *P* values < 0.05 were considered statistically significant.

## 3 Results

### 3.1 Exploring the differences between ibrutinib‐sensitive and ‐resistant CLL cells through integrated multi‐omics

**Fig. 1:**
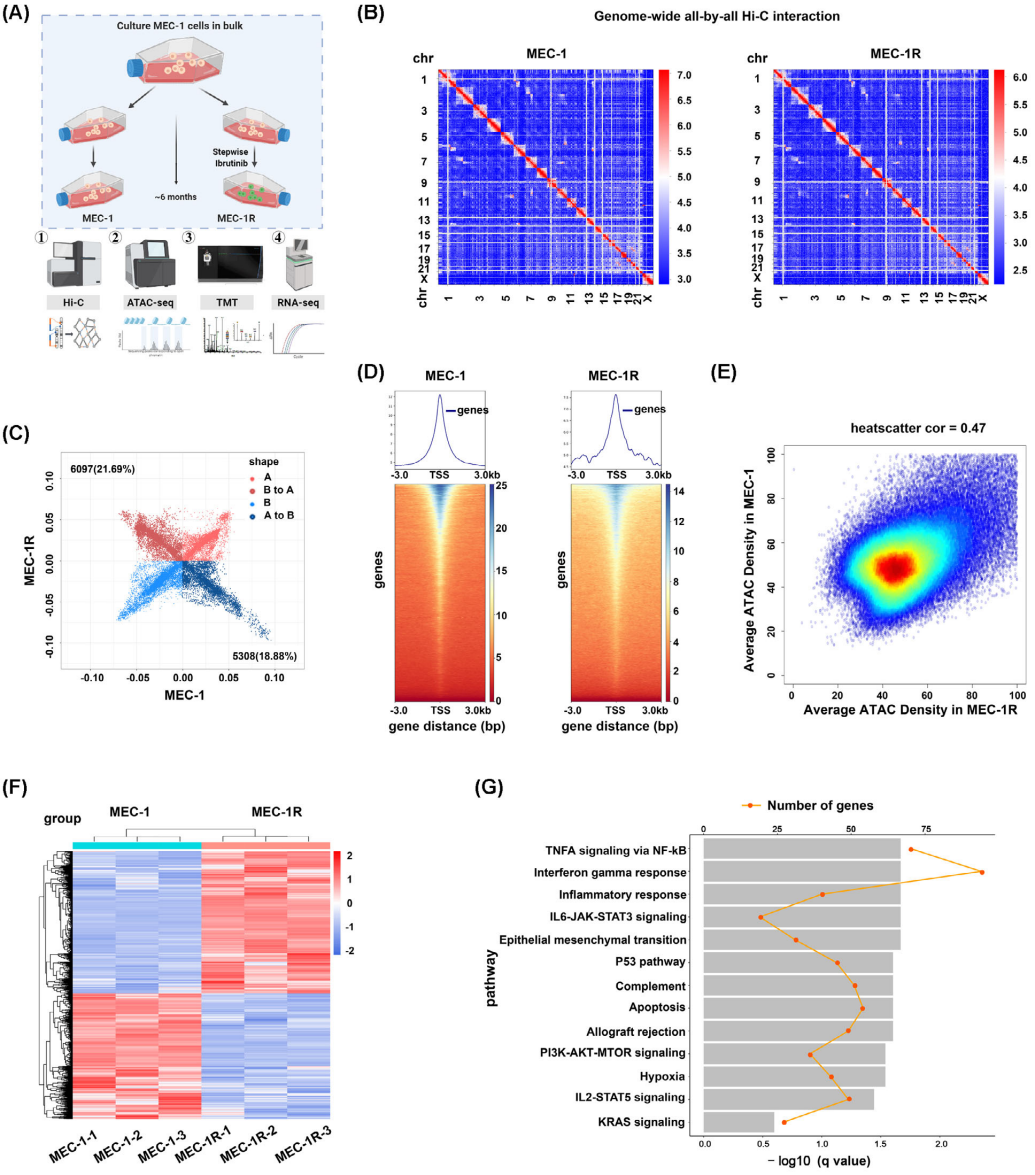
Study of ibrutinib‐resistant cells through integrated multi‐omics profiling. (A) Schematic procedure of the establishment of ibrutinib‐resistant cells from the CLL cell line MEC‐1 and the subsequently employed multi‐omics analyses. (B) Whole‐genome Hi‐C heatmaps of parental MEC‐1 cells (left) and ibrutinib‐resistant MEC‐1R cells (right). Chromosomes are stacked from the top left to the bottom right in order (from chr1 to chrX) (*N* = 3). (C) Scatter chart showing the compartment changes between MEC‐1 and MEC‐1R cells (*N* = 3). (D) Heatmaps of regions that are differentially accessible between MEC‐1 and MEC‐1R. (E) Heatscatter showing the correlation between MEC‐1 and MEC‐1R cells based on ATAC‐seq data. (F) Heatmap for up‐ and down‐regulated genes analysed by RNA‐seq. (G) Gene set enrichment analysis (GSEA) of the activated hallmark pathways in ibrutinib‐resistant cells comparing the two clusters. Multi‐omics profiling was performed with three independent biological replicates of MEC‐1 and MEC‐1R cells. [Colour figure can be viewed at wileyonlinelibrary.com]

To investigate acquired resistance to ibrutinib in CLL cells, we generated MEC‐1R cells by exposing MEC‐1 cells to increasing concentrations of ibrutinib. Multi‐omics were then used to explore changes in MEC‐1R cells at the DNA, RNA and protein levels (Fig. 1A). Hi‐C libraries from three independent biological replicates of MEC‐1 and MEC‐1R cells were generated. The analysis of Hi‐C revealed a hierarchical chromatin conformation and self‐interacting pattern in CLL cells, with intra‐chromosomal interactions (visualized as darker boxes along the diagonal) notably more frequent compared with inter‐chromosomal interactions. In addition, the similar organization pattern along the heatmap diagonal in both the MEC‐1 and MEC‐1R cells was observed, suggesting that the basic structural units of the genome remained stable upon the ibrutinib‐resistant cells (Fig. S1A and Fig. 1B). Interactions between chromosomes are displayed via a Circos plot (Fig. S1B). A previous study [19] indicated two unique patterns of genomic interactions, representing the open (A‐type) and closed (B‐type) genomic compartments. To determine whether the subscale chromatin conformations changed in ibrutinib‐resistant cells, we performed compartment classification, demonstrating that 18.88% of the genome exhibited a compartment pattern change from A to B, whereas 21.69% of the genome exhibited a change from B to A (Fig. 1C). This result indicated that although genomic compartmentalization is generally stable, differences do exist in ibrutinib‐resistant cells. It is well‐known that abnormalities on chromosome 11 (chr11) and chromosome 12 (chr12) are common in patients with CLL [24, 25, 26]. Interestingly, we found significant enrichment of genomic regions on chr11 and chr12 that switched to the A‐type compartment in ibrutinib‐resistant cells from the B‐type compartment observed in parental cells which suggested a higher frequency of open compartments on the two chromosomes (Fig. S1C). Compartments are composed of 100 kb to 1 Mb scale topologically associating domains (TADs), and intra‐TAD interactions are notably more frequent than inter‐TAD interactions [27]. Despite the differences in chromosomal structure and changes in compartmentalization, our results revealed that the TAD boundaries were consistent between ibrutinib‐resistant cells and parental cells (Fig. S1D,E).

Genomic changes often result from disruption of the chromatin conformation and the subsequent alteration of chromatin accessibility [28]. To test whether global chromatin accessibility was affected in ibrutinib‐resistant cells, we profiled the genome‐wide chromatin accessibility using ATAC‐Seq. The ATAC‐seq signal was significantly enriched at −1 to +1 kb from transcription start sites (Fig. 1D). In general, the distribution of chromatin accessibility remained relatively stable in ibrutinib‐resistant cells with a higher correlation coefficient (Fig. 1E). Subsequently, RNA‐seq analyses were performed to examine the transcriptional profile results from the changes of chromatin accessibility in the two groups. As a result, 1354 up‐regulated genes and 1195 down‐regulated genes were found (Fig. 1F). Gene set enrichment analysis (GSEA) of altered genes was then performed to explore relative pathways between the two groups. Ibrutinib‐resistant cells exhibited differential expression in genes related to TNF‐α signalling via NF‐κB, IL6‐JAK‐STAT3 signalling, PI3K‐AKT‐mTOR signalling and IL2‐STAT5 signalling activation (Fig. 1G). TMT quantitative proteomics analysis was further applied to compare changes at the protein level between the two groups. A total of 5640 proteins were identified, 269 of which showed opposite trends. The differential proteins obtained are presented in the form of clustered heatmaps in Fig. S1F. Taken together, these results demonstrated that ibrutinib‐resistant cells exhibited changes at the DNA, RNA and protein levels.

### 3.2 PAK1 and BCAT1 expression levels are up‐regulated in ibrutinib‐resistant CLL cells

**Fig. 2:**
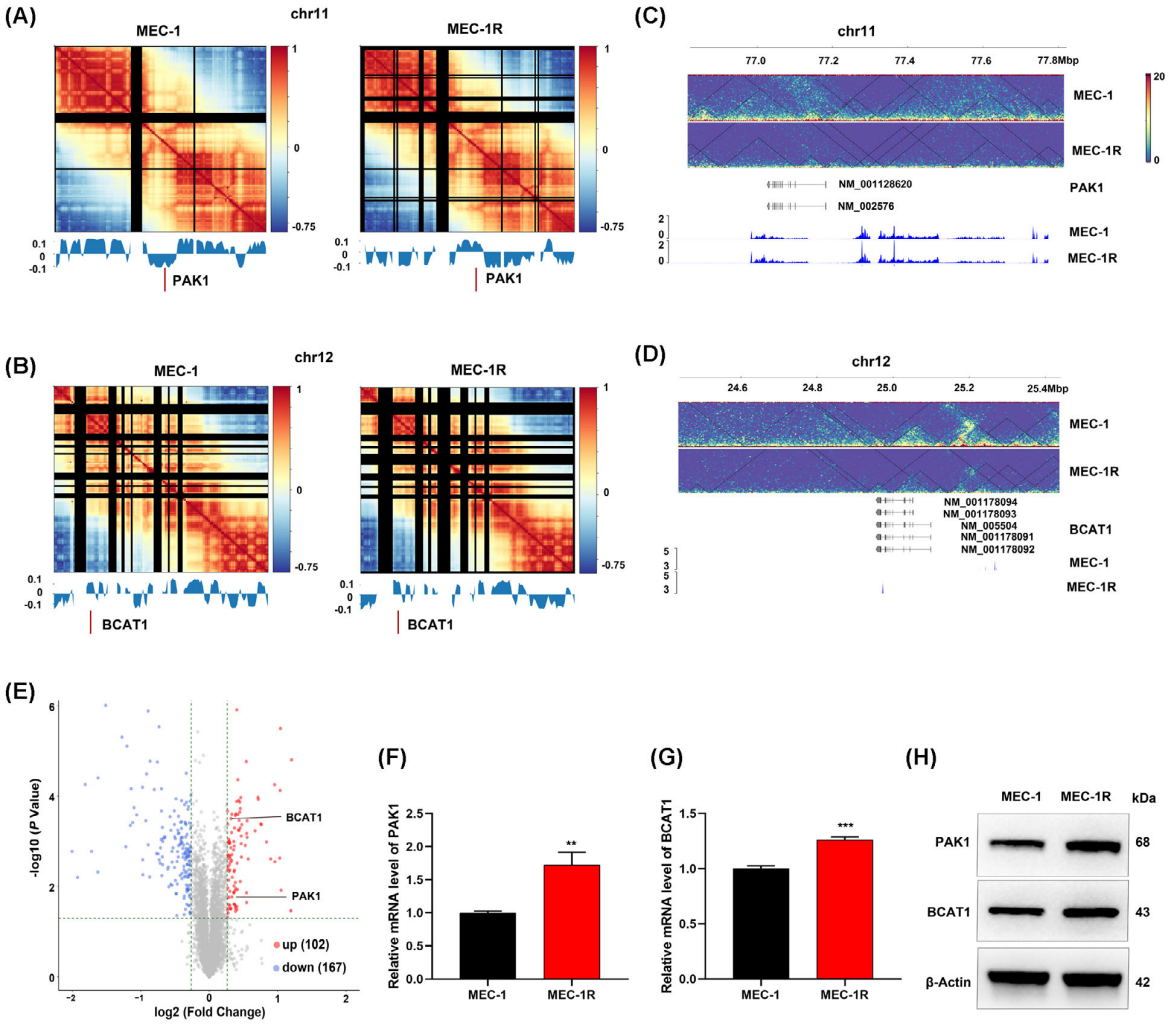
Dysregulation of PAK1 and BCAT1 in ibrutinib‐resistant cells. (A) Correlation map of chromosome 11 at a resolution of 1 m. Lower panels indicate bins that switched compartments. The indicated regions (red dashes) represent PAK1 loci. (B) Correlation map of chromosome 12 at a resolution of 1 m. Lower panels indicate bins that switched compartments. The indicated regions (red dashes) represent BCAT1 loci. Matrix of normalized differences in correlation coefficients between MEC‐1 and MEC‐1R cells for chromosome 11 (C) and chromosome 12 (D). Lower panels show the RNA‐seq analysis of the two groups. (E) Volcano plot comparing the expression fold changes of proteins between MEC‐1 and MEC‐1R cells based on TMT data. Multi‐omics profiling was performed with three independent biological replicates of MEC‐1 and MEC‐1R cells. (F–H) RT‐qPCR and western blot analysis showed the higher level of PAK1 and BCAT1 in MEC‐1R cells compared to MEC‐1 cells (*N* = 3) (mean ± SD). **P < 0.01and ***P < 0.001 by Student's *t* test. [Colour figure can be viewed at wileyonlinelibrary.com]

The integrated results of chromatin conformation, transcriptomics and proteomics revealed 110 dysregulated genes in ibrutinib‐resistant CLL cells (Fig. S1G and Table S1), among which 33 synchronously up‐regulated and 43 down‐regulated. PAK1 (p21‐activated kinase 1), located on chr11, particularly attracted our attention. Our Hi‐C data suggested the significant transformation of A‐to‐B compartments on chr11 and chr12. Of note, the PAK1 locus was subject to alteration from a B to an A compartment type which explained the up‐regulation of PAK1 in ibrutinib‐resistant cells (Fig. 2A). BCAT1 (branched chain amino acid transaminase 1), located on chr12, exhibited no significant change between A and B compartments (Fig. 2B). Data from the combined analysis integrating Hi‐C and RNA‐seq are shown in Fig. 2C,D. Meanwhile, TMT analysis also verified the higher expression of PAK1 and BCAT1 in ibrutinib‐resistant cells (Fig. 2E). In addition, our RT‐qPCR and western analysis confirmed these results (Fig. 2F–H).

### 3.3 PAK1 and BCAT1 expression levels are positively correlated and closely related to the prognosis of CLL patients

**Fig. 3:**
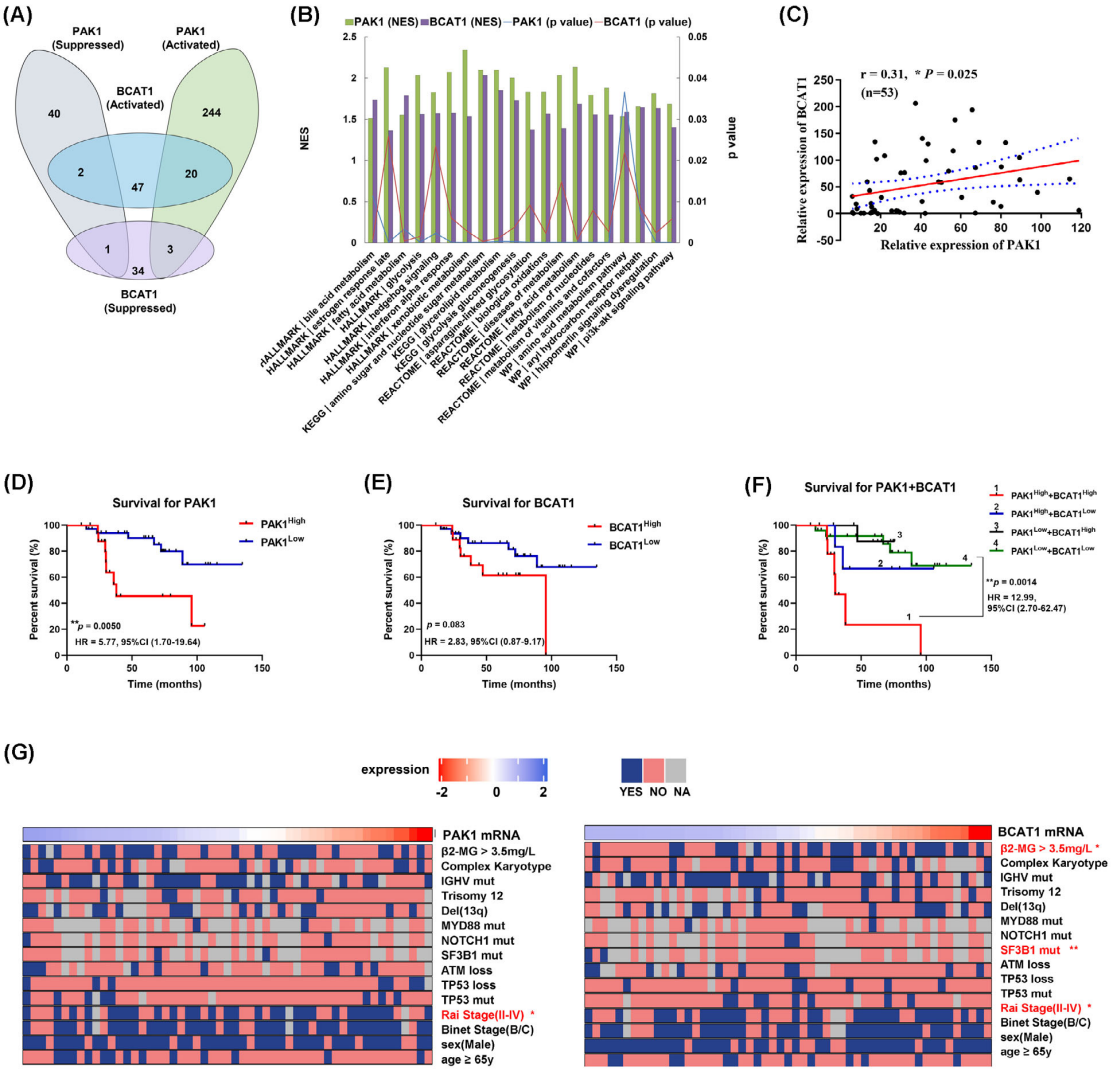
Correlation between BCAT1 and PAK1 expression and their prognostic value. (A) GSEA terms of the genes whose expression are positively correlated with PAK1 and BCAT1 expression in CLL from RNA‐seq datasets. Twenty signalling pathways were activated both in PAK1 and BCAT1 related genes and two were suppressed. (B) The 20 pathways that activated in PAK1‐ and BCAT1‐related gene sets. NES, normalized enrichment score. (C) Correlation between PAK1 and BCAT1 levels in CLL patients based on bulk RNA‐seq data. Pearson's correlation coefficient values (*r*) and *P* values are indicated. Kaplan–Meier analysis of the correlation between PAK1 expression (D), BCAT1 expression (E) or their combined expression (F) and overall survival (OS) in 53 patients with CLL. Log rank tests were used to determine statistical significance. (G) Univariate analysis to identify factors corresponding to the expression of PAK1 and BCAT1. Chi‐squared test, Pearson's test and Wilcoxon–Mann–Whitney test were used according to the types of data. *P < 0.05, **P < 0.01, ***P < 0.001. [Colour figure can be viewed at wileyonlinelibrary.com]

To determine the genes expression profiles in CLL patients, we employed RNA‐seq on 53 clinical treatment‐naïve CLL samples. PAK1 was previously reported to regulate cancer cell metabolism and proliferation [29, 30] and BCAT1 is implicated in cancer metabolic reprogramming, tumour metastasis and drug resistance [31, 32, 33]. Given that the expression levels of both PAK1 and BCAT1 were up‐regulated in ibrutinib‐resistant cells, we performed GSEA to explore the enriched pathways in CLL. A total of 2227 positively associated genes and 1744 negatively associated genes were identified for PAK1, whereas 1110 positively and 424 negatively associated genes were identified for BCAT1 (*P* < 0.05). Subsequent functional enrichment analysis revealed that PAK1 and BCAT1‐related genes were associated with glycolysis, fatty acid oxidation and PI3K‐AKT‐mTOR signalling, consistent with previous reports. Strikingly, among the gene sets enriched in BCAT1‐related genes, approximately 20 overlapped with those for PAK1 (Fig. 3A,B). These observations indicated that PAK1 and BCAT1 are involved in many common pathways. Furthermore, correlation analysis revealed a significant positive correlation between the expression of the two genes (Fig. 3C).

Next, clinical information was collected to assess the prognostic value of PAK1 and BCAT1 in CLL patients. Kaplan–Meier analysis revealed that the increased expression of PAK1 was associated with worse overall survival, whereas no significant difference in survival was observed between patients with different BCAT1 expression (Fig. 3D,E). However, when combining BCAT1 with PAK1, the prognostic value was greatly enhanced (Fig. 3F). The results prompted us to explore their correlation with the prognostic factors. The clinical characteristics of 53 patients enrolled in the cohort are summarized in Fig. 3G and Table S2. Higher expression of PAK1 was significantly correlated with high‐risk Rai stages (II–IV, *P* = 0.04). BCAT1 expression was significantly correlated with elevated beta‐2‐microglobulin levels (≥ 3.5 mg·L^−1^, *P* = 0.015), advanced Rai stage (*P* = 0.040) and SF3B1 mutation status (*P* = 0.0092). Taken together, our results revealed that PAK1 is associated with the outcome in patients with CLL and its combination with BCAT1 helps to improve the prognostic accuracy.

### 3.4 PAK1 regulates the metabolism of CLL cells and affects the sensitivity to ibrutinib

**Fig. 4:**
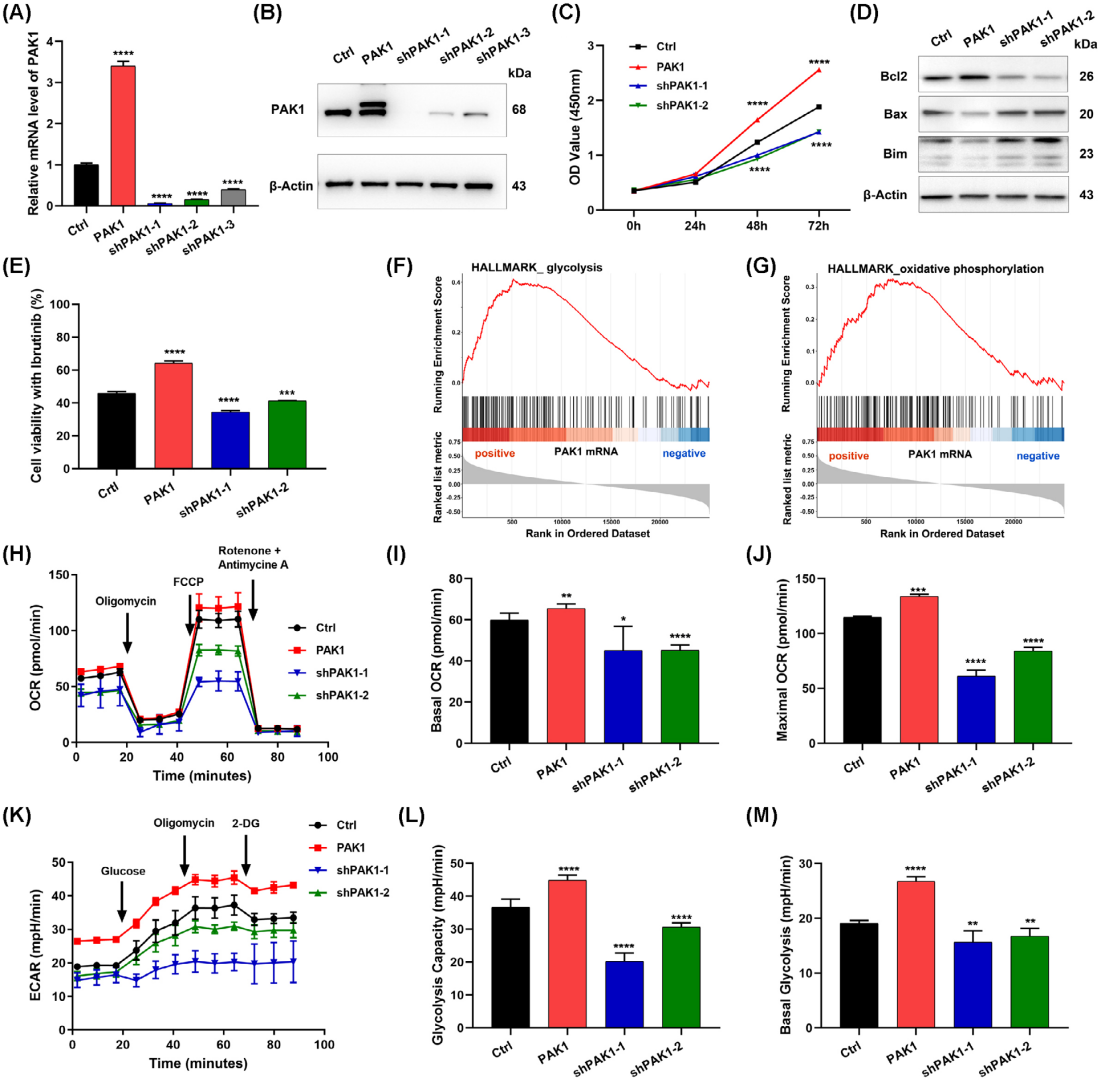
PAK1 promotes cell proliferation, metabolic reprogramming and mediates ibrutinib resistance. (A) RT‐qPCR to verify the expression of PAK1 in MEC‐1 cells transfected with shRNAs (sh‐PAK1) or an overexpression vector (PAK1) compared to control cells (Ctrl; *N* = 3; mean ± SD). (B) Protein expression of PAK1 after transfection with shRNAs or an overexpression vector. All experiments were performed in triplicate. The *t* test was used to estimate the *P* value. (C) CCK8 assays showed the higher proliferation ability of PAK1 cells and lower of sh‐PAK1 cells compared to Ctrl group (*N* = 3; mean ± SD). (D) The levels of proapoptotic and antiapoptosis protein detected using western blot. All experiments were performed in triplicate. (E) Sensitivity of MEC‐1 cells to ibrutinib based on different PAK1 expression analysed by CCK8 assay (*N* = 3; mean ± SD). (F, G) GSEA of genes related to PAK1 based on the RNA‐seq data and glycolysis and oxidative phosphorylation were positively associated with the expression of PAK1. (H) Seahorse metabolic analysis of the OCR in MEC‐1 cells with PAK1 overexpression and knockdown. (I) Basal OCR levels determined before oligomycin injection and (J) maximal OCR levels determined after FCCP injection were analysed. OCR, oxygen consumption; FCCP, carbonyl cyanide *p*‐[trifluoromethoxy]‐phenyl‐hydrazone. (K) ECAR of MEC‐1 cells after overexpressing and knocking down PAK1. (L) Glycolysis rate was analysed after the injection of glucose and (M) glycolytic capacity was detected after the injection of oligomycin. ECAR, extracellular acidification rate. Data are presented as the mean ± SD of three independent experiments. *P < 0.05, **P < 0.01, ***P < 0.001, ****P < 0.0001. [Colour figure can be viewed at wileyonlinelibrary.com]

To gain further insight into the role of PAK1 in CLL, functional assays were performed in MEC‐1 cells. Lentivirus‐mediated PAK1 overexpression was employed for gain‐of‐function assays. PAK1 mRNA and protein overexpression was confirmed via RT‐qPCR and western blot analysis respectively. Three lentivirus‐mediated RNAi vectors against PAK1 demonstrated effective silencing of PAK1 expression at both the mRNA and protein levels. shPAK1‐1 and shPAK1‐2 exhibited higher knockdown efficacy and were thus used in subsequent experiments (Fig. 4A,B). PAK1 overexpression significantly promoted cell proliferation and its knockdown markedly suppressed cell viability (Fig. 4C). Moreover, PAK1 suppressed the expression of pro‐apoptotic proteins (Bax, Bim) and promoted the expression of the anti‐apoptosis protein Bcl‐2 (Fig. 4D). To determine the impact of PAK1 on ibrutinib sensitivity, cells were exposed to various concentrations of ibrutinib for 48 h. Cell viability assays indicated that the depletion of PAK1 increased ibrutinib sensitivity. In contrast, PAK1 overexpression suppressed drug sensitivity relative to the control cells (Fig. 4E).

The metabolism of tumour cells is often very different from that of normal cells. CLL cells have been reported to be dependent on oxidative phosphorylation for their energy supply [34, 35, 36]. GSEA indicated a strong association of PAK1‐related genes with glycolysis (Fig. 4F) and oxidative phosphorylation (Fig. 4G). Thus, we evaluated the effect of PAK1 on oxygen consumption and glycolysis in CLL. Mitochondrial respiration and glycolytic metabolism were assessed via measurement of the OCR and ECAR respectively. PAK1 overexpression led to an overtly elevated basal OCR and significantly higher maximum OCR compared with those of controls. In contrast, PAK1 knockdown decreased OCR at both the basal and maximal level (Fig. 4H–J). ECAR data indicated that the knockdown of PAK1 via two shRNAs reduced the overall glycolytic flux. Glycolysis and glycolytic capacity were all significantly reduced by PAK1 knockdown, whereas the overexpression of PAK1 caused the opposite results (Fig. 4K–M). In summary, these data suggested that PAK1 positively regulates glycolysis and oxidative phosphorylation in CLL cells.

### 3.5 PAK1 correlates with mTORC1 signalling and the clinical outcome of CLL

**Fig. 5:**
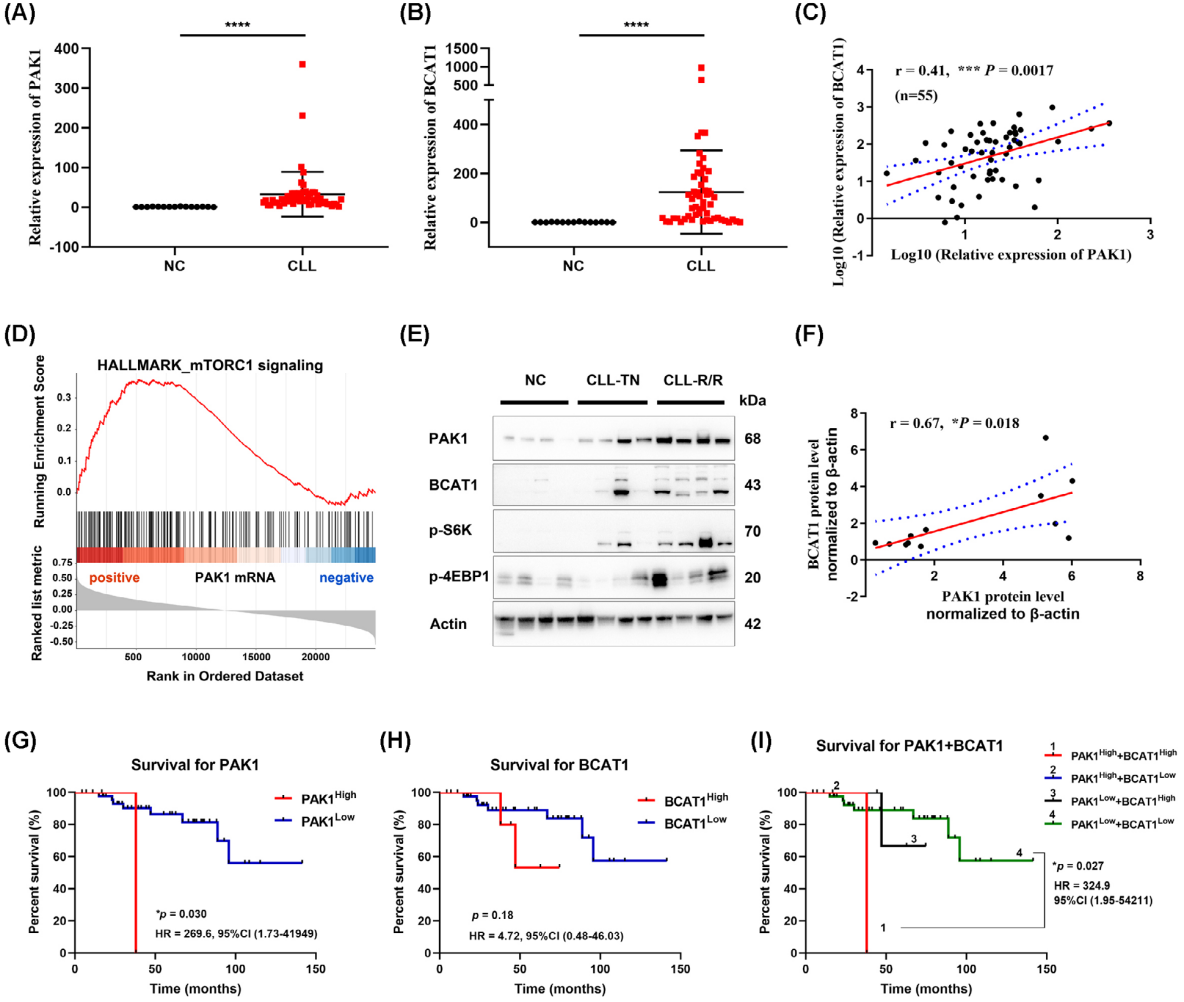
Expression and clinical prognostic value of PAK1 and BCAT1 in CLL patients. (A) Expression of *PAK1* in CLL patients (*n* = 15, mean ± SD) compared to normal controls (*n* = 55, mean ± SD), as determined via RT‐qPCR. NC, normal controls (CD19^+^ B cells from healthy volunteers). *P* values were calculated using the two‐tailed *t*‐test statistical analysis. (B) BCAT1 mRNA levels in CLL patients compared to normal controls. *P* values were calculated using the two‐tailed *t*‐test statistical analysis, error bars indicated SD. (C) Scatter plots showing the correlation between PAK1 and BCAT1 mRNA expression (*N* = 55). (D) GSEA of PAK1‐related genes. (E) The protein levels of PAK1, BCAT1 and mTORC1 signalling molecules in CLL patients and normal controls. TN, treatment naive; R/R, refractory or relapsed. All experiments were performed in triplicate. (F) The quantitative analysis of protein bands of PAK1 and BCAT1 and correlation coefficient was calculated (*N* = 12). Kaplan–Meier analysis of the correlation between PAK1 expression (G), BCAT1 expression (H) or their combined expression (I) and overall survival (OS) in the validated cohort of 53 CLL patients (*N* = 50; survival data of three patients was now available), Log rank tests were used to determine statistical significance. *P < 0.05, ***P < 0.001, ****P < 0.0001. [Colour figure can be viewed at wileyonlinelibrary.com]

To verify the sequencing results and further explore the role of PAK1 in CLL, another larger cohort of B cells from healthy volunteers and PBMCs from CLL patients were collected. The mRNA levels of PAK1 were significantly higher in CLL patients (Fig. 5A). Up‐regulated expression of BCAT1 was also found compared to normal B cells (Fig. 5B). Consistent with the sequencing results, a positive relationship was observed between PAK1 and BCAT1 (Fig. 5C). As GSEA implicated that PAK1 and BCAT1 were both functionally enriched in mTORC1 signalling, the levels of related proteins were detected (Fig. 5D, Fig. S2A). The expression of PAK1 and BCAT1 along with mTORC1‐related proteins was increased in CLL patients compared with those in the control group, especially in refractory or relapsed CLL patients (Fig. 5E, Table S3). In addition, Spearman's correlation analysis of PAK1 and BCAT1 protein levels revealed a significant correlation (Fig. 5F). Kaplan–Meier survival analysis based on RT‐qPCR data further confirmed the prognostic value of PAK1 and BCAT1. Higher PAK1 expression was associated with shorter overall survival (OS) and BCAT1, though has no correlation with OS, its combination with PAK1 assisted in differentiating CLL patients with poor outcomes (Fig. 5G–I). These results were consistent with the sequencing results regarding the prognostic value of PAK1 and BCAT1 expression in CLL patients.

### 3.6 PAK1 inhibitor IPA‐3 exerts anti‐tumour activity in CLL cells through mTORC1 signalling

**Fig. 6:**
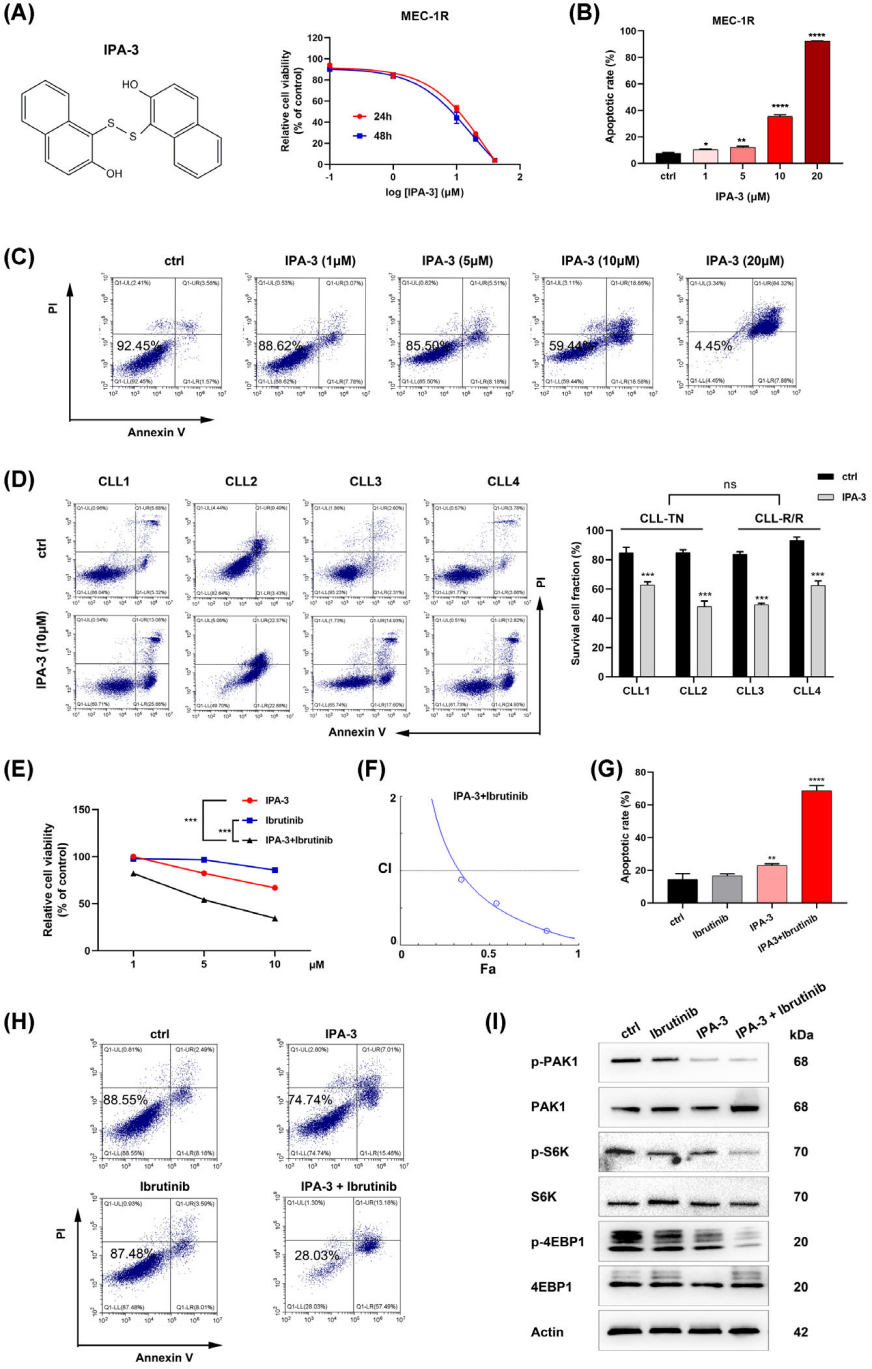
The PAK1 inhibitor IPA‐3 exerts anti‐tumour effects in ibrutinib‐resistant cells. (A) IPA‐3‐mediated inhibition of growth in parental cells and ibrutinib‐resistant cells. Left: The chemical structure of IPA‐3. Right: Growth curves of MEC‐1R cells under treatment with different concentrations of IPA‐3 detected by CCK8 assay (IPA‐3, dose range: 0–40 μm, IC50: 21.83 μm). (B, C) Apoptosis analysis of MEC‐1R cells treated with IPA‐3 (*N* = 3; mean ± SD). The two‐way ANOVA test was used to estimate the *P* value. (D) Apoptosis analysis of CLL primary cells treated with IPA‐3. CLL1 and CLL2 from treatment‐native patients; CLL3 and CLL4 from ibrutinib‐resistant CLL patients (*N* = 3; mean ± SD). The two‐way ANOVA test was used to estimate the *P* value. (E) CCK8 assay of ibrutinib‐resistant MEC‐1R cells treated with ibrutinib or ibrutinib in combination with IPA‐3 at the indicated doses for 48 h. The two‐way ANOVA test was used to estimate the *P* value. (F) Combination index (CI) calculated using compusyn software. (G, H) Apoptosis analysis of MEC‐1R cells treated with ibrutinib, IPA‐3 or both (*N* = 3; mean ± SD). The two‐way ANOVA test was used to estimate the *P* value. (I) Comparison of the inhibition of PAK1 and mTORC1 signalling in MEC‐1R cells exposed to IPA‐3 and/or ibrutinib. All experiments were performed in triplicate. Data are presented as the mean ± SD of three independent experiments. *P < 0.05, **P < 0.01, ***P < 0.001, ****P < 0.0001. [Colour figure can be viewed at wileyonlinelibrary.com]

To investigate the effect of PAK1 inhibitor IPA‐3 on CLL cells, CCK8 assays were conducted. IPA‐3 suppressed the proliferation of MEC‐1 in a dose‐dependent manner (Fig. S3A). Of note, IPA‐3 suppressed the viability of ibrutinib‐resistant cells (MEC‐1R) in the same manner (Fig. 6A). Annexin V and PI staining was then performed to investigate whether IPA‐3 triggered cell apoptosis. Incubation of MEC‐1R cells with higher doses of IPA‐3 led to a higher percentage of apoptotic cells, suggesting that cells underwent apoptosis under IPA‐3 treatment (Fig. 6B,C). Similar results were found for MEC‐1 and JVM‐3 CLL cells (Fig. S3B–F). In addition, primary cells from two treatment‐native and two ibrutinib‐resistance CLL patients were used to test the killing role of IPA‐3. Inspiringly, IPA‐3 induced the apoptosis of these cells basically equally (Fig. 6D). Meanwhile, the BCAT1 inhibitors gabapentin and BCATc inhibitor 2 [37, 38] were ineffective in CLL cells (Fig. S4A,B). However, a synergistic effect was found with the combination of IPA‐3 and BCAT1 inhibitors (Fig. S4C–H and Table S4).

As PAK1 up‐regulation was associated with ibrutinib resistance, we wondered whether targeting PAK1 with IPA‐3 could recover the sensitivity of MEC‐1R cells to ibrutinib. As expected, the combined use of IPA‐3 and ibrutinib exerted potent cell growth inhibition (Fig. 6E) and the CI indicated their synergistic effect (Fig. 6F). And apoptotic assays confirmed this result (Fig. 6G,H). This effect was accompanied by a reduction in PAK1 and mTORC1 phosphorylation levels (Fig. 6F). These data demonstrated that IPA‐3 induced inhibition of PAK1 may facilitate enhancing the potency of ibrutinib and thus overcome resistance.

## 4 Discussion

Although ibrutinib has brought promising curative effects for CLL patients, drug resistance remains a major issue. Mutations of the active kinase domain on the BTK enzyme and CARD11 are reported as common factors of BTKi resistance in CLL [39, 40]. In this study, none of these mutations occurred during the establishment of ibrutinib‐resistant MEC‐1 cells (MEC‐1R), which was used as an *in vitro* model of acquired resistance. We further integrated multi‐omics profiling, including Hi‐C technology, for the analysis of MEC‐1 and MEC‐1R.

The spatial organization of chromatin is central to cellular homeostasis and the response to drug treatment [7]. Thus, alterations in chromatin structure may be involved in the occurrence of drug resistance; however, little is known regarding their significance in CLL and ibrutinib resistance. Herein, we explored chromatin spatial dynamics via Hi‐C analysis, which has not been studied in CLL to date. Our results demonstrated that chromosome 11 was one of the chromosomes altered in ibrutinib‐resistant cells. PAK1, which is located on chr11, was frequently overexpressed or excessively activated in almost all cancer types and is involved in nearly every stage of cancer progression [41]. From the analysis, we observed higher expression of PAK1 in ibrutinib‐resistant cells. Therefore, we further focused on the role of PAK1 in resistance to ibrutinib, which has not been previously reported in CLL. Through Hi‐C analysis, the oncogene PAK1 was observed located in a region where B‐to‐A compartment switching occurred in MEC‐1R cells. This alteration of compartments from close to open suggested the genes in the related region are possibly up‐regulated. Consistent with Hi‐C analysis, the results of ATAC‐seq, RNA‐seq and TMT revealed the up‐regulation of PAK1 in ibrutinib‐resistant CLL cells. Previous studies have demonstrated the significance of PAK1 in multiple cancer types, which participated in cell survival and drug resistance [42]. For example, PAK1 was reported to mediate the resistance to PI3 kinase inhibitors in lymphoma [43]. In this study, we explored and confirmed the oncogenic role of PAK1 in CLL for the first time. It is well‐known that energy demands are increased for tumour cells, and carbohydrate metabolism, lipid metabolism and oxidative phosphorylation all play crucial roles in CLL [35]. Here, we confirmed that PAK1 regulates glycolysis and oxidative phosphorylation in CLL cells. The metabolic changes caused by PAK1 might contribute to ibrutinib resistance. The expression of BCAT1 was also evaluated in CLL patients and ibrutinib‐resistant cells as BCAT1 is known to play roles in metabolic reprogramming, disease progression and drug resistance [37, 38, 44]. We found that BCAT1 expression was positively correlated with PAK1 and including both in a prognostic model had greater predictive value than using them alone. Based on the association between PAK1 and BCAT1, we questioned whether there might be a regulatory relationship between these two genes. RT‐qPCR analysis revealed that silencing PAK1 did not alter the expression of BCAT1 or vice versa (data not shown), indicating that these two molecules might function through independent downstream signalling pathways. Thus, further study of the mechanism underlying their synergy is warranted.

As the incidence of acquired ibrutinib resistance increases during long‐term ibrutinib treatment, patients may benefit from the exploration of new therapeutic targets and drug combination strategies [45]. In this study, we evaluated the efficacy of the PAK1 inhibitor IPA‐3 as well as the BCAT1 inhibitors gabapentin and BCATc inhibitor 2 in CLL. Based on the current findings, IPA‐3 exhibited efficacy against ibrutinib‐resistant CLL cells *in vitro* and with ibrutinib synergistically induced apoptosis of tumour cells. As a regulator of the mTORC1 signalling pathway, the PAK1 inhibitor showed potential therapeutic value for CLL patients with ibrutinib resistance. In contrast, BCAT1 inhibitors did not exhibit significant anti‐tumour efficacy in CLL. However, the combined use of IPA‐3 and BCAT1 inhibitors exhibited a synergistic effect. Collectively, this result implies the promising roles of PAK1 and its inhibitor IPA‐3 in CLL. Nonetheless, the curative efficiency needs to be explored *in vivo* with animal models in the future.

## 5 Conclusion

In conclusion, our results provide a genome‐wide view of alterations in 3D chromatin organization between ibrutinib‐resistant and parental CLL cells. To the best of our knowledge, this is the first work to explore drug resistance in CLL from the perspective of 3D genome organization and the first study to reveal the roles of PAK1 in CLL. And this result provides promising therapeutic targets for overcoming ibrutinib resistance. In particular, the treatment of CLL patients with a combination of IPA‐3 and ibrutinib may improve clinical outcomes.

## Conflict of interest

The authors declare no conflict of interest.

## Author contributions

HJ, JL and ZW conceptualized the study. ZW, LW and LF led the experimental design and wrote the manuscript. ZW, XZ, HT, DG, XL, YL and JW performed the experiments. SQ, YX, HZ and LW provided human samples and corrected the manuscript. WX supervised the study. All authors analysed and interpreted the data.

### Peer review

The peer review history for this article is available at https://publons.com/publon/10.1002/1878‐0261.13281.

## Supporting information


**Fig. S1.** Multi‐omics profiling of MEC‐1 and MEC‐1R cells.

**Fig. S2.** Analysis of PAK1‐related genes.

**Fig. S3.** Analysis of IPA‐3‐induced apoptosis.

**Fig. S4.** Cell growth suppression by BCAT1 inhibitor and its combination with IPA‐3.

**Table S1.** Statistics test methods of mutli‐omics profiling and integrated results of chromatin conformation, transcriptomics and proteomics.

**Table S2.** Clinical characteristics of 53 patients enrolled in the RNA sequencing.

**Table S3.** Detailed characteristics of eight CLL patients.

**Table S4.** CIs for different levels of growth inhibition (fraction affected) calculated using the compusyn software.

**Supinfo.** Supplementary material.

## Data Availability

The datasets and computer code produced in this study are available in the following databases: Multi‐omics sequence of MEC‐1 cells and MEC‐1R cells including Hi‐C, ATAC‐seq, RNA‐seq and TMT: National Center for Biotechnology Information Sequence Read Archive (SRA), PRJNA744093. RNA sequencing of 53 CLL patients: PRJNA762572 (https://dataview.ncbi.nlm.nih.gov/object/PRJNA762572?reviewer=r2dipqp997m1j744v1s66vra83).
