# Supplementary figures and images for: Exploring the significance of PAK1 through chromosome conformation signatures in ibrutinib‐resistant chronic lymphocytic leukaemia
Source: Mol Oncol. 2022 Jul 22;16(16):2920–35. doi: 10.1002/1878-0261.13281 (PMC9394240; doi:10.1002/1878-0261.13281)

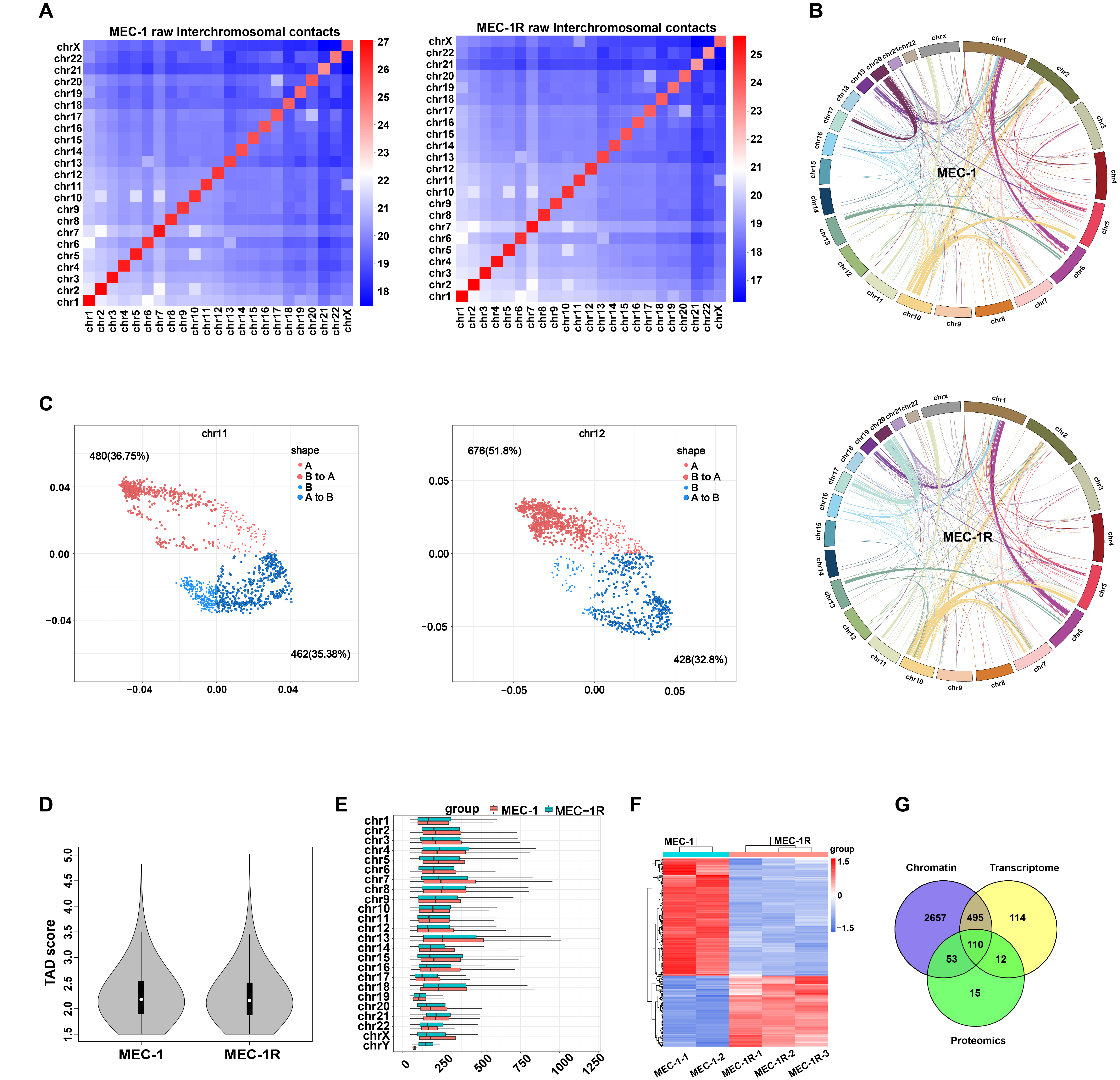

Supplement: Supplementary file 1 — Fig. S1. Multi‐omics profiling of MEC‐1 and MEC‐1R cells. [file MOL2-16-2920-s008.jpg]

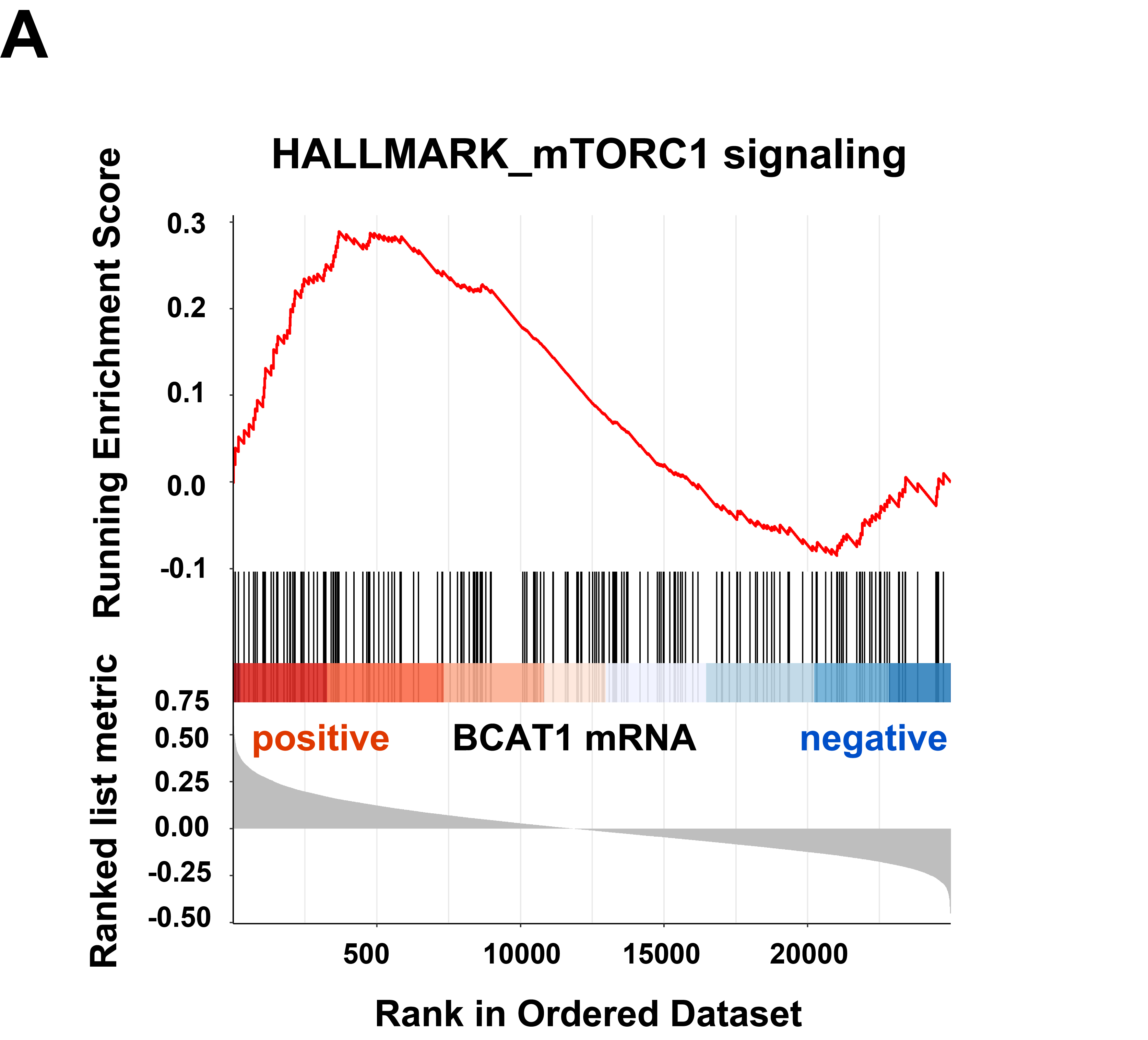

Supplement: Supplementary file 2 — Fig. S2. Analysis of PAK1‐related genes. [file MOL2-16-2920-s006.jpg]

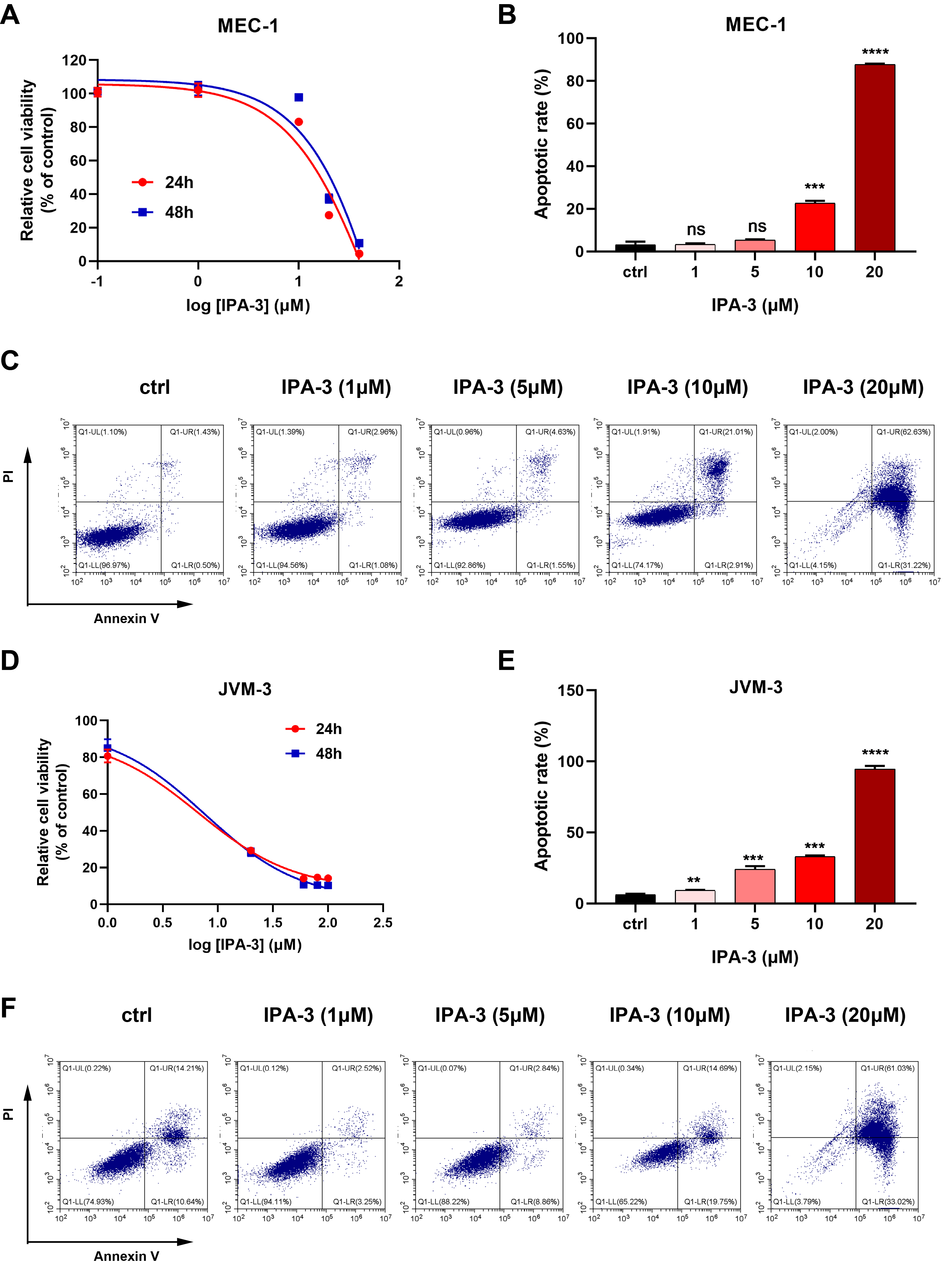

Supplement: Supplementary file 3 — Fig. S3. Analysis of IPA‐3‐induced apoptosis. [file MOL2-16-2920-s009.jpg]

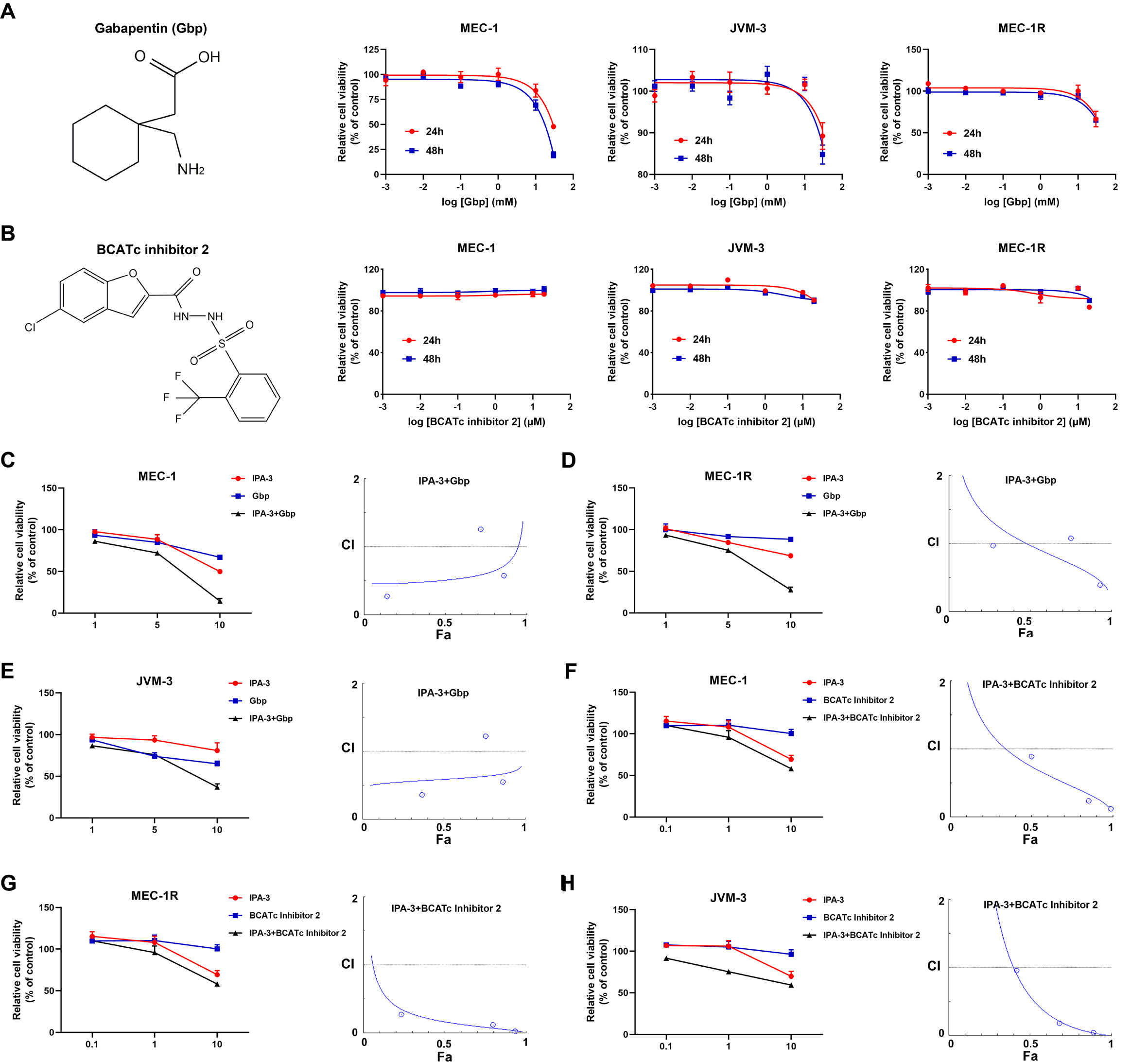

Supplement: Supplementary file 4 — Fig. S4. Cell growth suppression by BCAT1 inhibitor and its combination with IPA‐3. [file MOL2-16-2920-s003.jpg]
