# Supplementary material for: Exploring the significance of PAK1 through chromosome conformation signatures in ibrutinib‐resistant chronic lymphocytic leukaemia
Source: Mol Oncol. 2022 Jul 22;16(16):2920–35. doi: 10.1002/1878-0261.13281 (PMC9394240; doi:10.1002/1878-0261.13281)
Supplement: Supplementary file 9 — Supinfo. Supplementary material. [file MOL2-16-2920-s004.docx]

**Supplementary Figures**

**Fig. S1. Multi-omics profiling of MEC-1 cells and MEC-1R cells A.** Raw whole-genome Hi-C heatmaps of parental cells MEC-1 (upper) and ibrutinib-resistant cells MEC-1R (lower). Chromosomes are stacked from the top left to bottom right in order (from chr1 to chrX). **B.** Circos diagram of the trans-interacting fragments, with the periphery showing different chromosomes in the genome; the curve in the Circos indicates the location of the 1000 binpairs with the strongest interaction. **C.** Scatter chart showing the compartment changes between MEC-1 and MEC-1R cells in chr11 and chr12. **D.** Violin plot showing the size of TAD in MEC-1 and MEC-1R cells. **E.** Distribution of TAD on different chromosomes between MEC-1 and MEC-1R groups. **F.** The heatmap for up-and down-regulated proteins detected via TMT. **G.** Venn diagram of altered gene expression profiles based on ATAC-seq, RNA-seq, and TMT data.

**Fig. S2. Analysis of PAK1 related genes. A.** GSEA of BCAT1-related genes revealed that BCAT1 expression was significantly correlated with mTORC1 signaling.

**Fig. S3. Analysis of IPA-3-induced apoptosis. A.** CCK8 analysis detected the growth inhibition effect of MEC-1 cells treated with IPA-3 (IPA-3, dose range: 0–40 μM, IC50: MEC-1, 21.13 μM). **B-C.** Apoptosis analysis of MEC-1 cells treated with IPA-3. D. Growth curve of JVM-3 treated with IPA-3. **E-F.** Apoptosis analysis of JVM-3 treated with IPA-3. Annexin V-FITC/propidium iodine (PI) was used to detect apoptosis. Results were analyzed via flow cytometry. Data are presented as the mean ± SD of three independent experiments. **P* < 0.05, ***P* < 0.01, ****P* < 0.001, *****P* < 0.0001.

**Fig. S4. Cell growth suppression by BCAT1 inhibitor and its combination with IPA-3. A.** BCAT1 inhibitor Gabapentin suppressed the growth of MEC-1, MEC-1R, and JVM-3 cells. **B.** BCAT1 inhibitor BCATc inhibitor 2 suppressed the growth of MEC-1, MEC-1R, and JVM-3 cells. **C-H.** Combination analyses performed via the median-effect method. Cells were exposed to PAK1 and BCAT1 inhibitors at a constant concentration ratio of 1:1 for 24 hours. CIs for different levels of growth inhibition (fraction affected) were calculated using the CompuSyn software.

**Supplementary Tables**

**Table S1.** Statistics test methods of mutli-omics profiling and integrated results of chromatin conformation, transcriptomics, and proteomics.

**Table S2.** Clinical characteristics of 53 patients enrolled in the RNA sequencing.

**Table S3.** Detailed characteristics of 8 CLL patients.

**Table S4.** CIs for different levels of growth inhibition (fraction affected) calculated using the CompuSyn software.
